# Diet in Intestinal Fibrosis: A Double-Edged Sword

**DOI:** 10.3390/nu13093148

**Published:** 2021-09-09

**Authors:** Rachel Marion-Letellier, Mathilde Leboutte, Asma Amamou, Maitreyi Raman, Guillaume Savoye, Subrata Ghosh

**Affiliations:** 1 UNIROUEN, INSERM UMR 1073 Nutrition, Inflammation and Gut-Brain Axis, Normandie University, 76183 Rouen, France; mathilde.leboutte@etu.univ-rouen.fr (M.L.); guillaume.savoye@chu-rouen.fr (G.S.); 2 Institute for Research and Innovation in Biomedicine (IRIB), UNIROUEN, 76183 Rouen, France; 3 APC Microbiome Ireland, Biosciences Building, University College Cork, Cork, Ireland; AAmamou@ucc.ie (A.A.); SubrataGhosh@ucc.ie (S.G.); 4 Division of Gastroenterology, University of Calgary, Calgary, AB T2N 4N1, Canada; mkothand@ucalgary.ca; 5 Department of Community Health Sciences, University of Calgary, Calgary, AB T2N 4N1, Canada; 6 Department of Gastroenterology, Rouen University Hospital, 76031 Rouen, France

**Keywords:** fibrosis, Crohn’s disease, diet, gut microenvironment

## Abstract

The natural history of inflammatory bowel diseases, especially Crohn’s disease, is frequently complicated by intestinal fibrosis. Because of the lack of effective treatments for intestinal fibrosis, there is an urgent need to develop new therapies. Factors promoting intestinal fibrosis are currently unclear, but diet is a potential culprit. Diet may influence predisposition to develop intestinal fibrosis or alter its natural history by modification of both the host immune response and intestinal microbial composition. Few studies have documented the effects of dietary factors in modulating IBD-induced intestinal fibrosis. As the mechanisms behind fibrogenesis in the gut are believed to be broadly similar to those from extra-intestinal organs, it may be relevant to investigate which dietary components can inhibit or promote fibrosis factors such as myofibroblasts progenitor activation in other fibrotic diseases.

## 1. Intestinal Fibrosis

Inflammatory bowel diseases (IBD) are relapsing systemic inflammatory diseases, mainly affecting the gastrointestinal tract. IBD occurs in people with susceptibility genes triggered by environmental factors. It leads to an exacerbated immune response associated with a gut dysbiosis. The natural history of IBD is frequently complicated by intestinal fibrosis and strictures formation. More than half of patients with Crohn’s disease (CD) develop intestinal fibrosis, especially when the ileum is involved (Montreal classification L1) [1,2]. We and others have previously shown that stricturing CD may not respond well to anti-inflammatory therapy such as anti-TNFα, the gold standard in IBD treatment [3]. Eighty percent of CD patients with intestinal fibrosis and strictures undergo resection, but it frequently recurs, leading to repeated surgeries. Chronic inflammation induces remodeling of the intestinal wall by a cascade of events from intestinal epithelial damages to angiogenesis and immune and mesenchymal cells activation [4]. There is currently no specific therapy to prevent or inhibit intestinal fibrosis and this therefore constitutes an unmet need in IBD.

### Western Diet

Environmental factors, especially diet, may influence predisposition to develop IBD or alter its course. Diet can target both the host immune response [5,6], and intestinal microbial composition. In addition, diet is a recurrent concern for IBD patients and most of IBD patients believe that diet may be a trigger of disease activity [5,7,8]. The IBD incidence is higher in Western countries [5] and this incidence continues to increase in the newly industrialized countries adopting the Western diet [7]. Diet plays a key role in controlling gut immune homeostasis. Factors promoting intestinal fibrosis are currently unknown, but diet is a potential culprit. Indeed, the Western diet promotes fibrosis in studies from other organs [8,9]. The Western diet is characterized by an insufficient intake of healthy foodstuffs and an excessive amount of saturated fats, sugar, and salt. Even IBD patients in remission have considerably distorted and unhealthy dietary intake leading to an increased risk of nutritional deficiencies [10]. Very recently, a prospective cohort study has demonstrated an association between ultra-processed food consumption and IBD risk [11].

## 2. Obesity

The incidence of obesity is increasing worldwide and we previously demonstrated increasing body weight over time from 1991 to 2008 in CD as evidenced by baseline data from 40 randomized clinical trials [12]. In addition, CD patients exhibited a higher clinical disease activity and duration over the same time period [12]. Adiposity may thus play a potential role in initiating and perpetuating intestinal inflammation. In addition, obesity is associated with chronic gut inflammation and visceral fat accumulation as observed in CD. Recently, visceral obesity was associated with adverse outcomes in severe CD patients [13] while IBD patients with weight loss after bariatric surgery had fewer complications [14].

**Figure 1:**
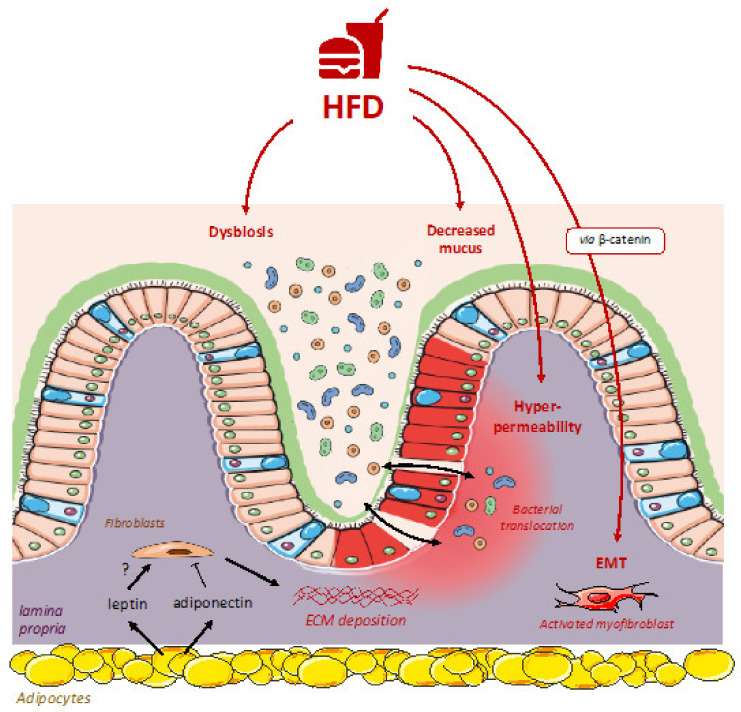
How high fat diets may contribute to intestinal fibrosis?

High-fat diets (HFD) have a significant impact on gut physiology and mucosal defenses (Figure 1). Thirty-days HFD is sufficient to alter the spatial distribution and composition of the microbiota [15]. Innate immunity is also altered with: (i) decreased antimicrobial peptide with (ii) a reduction in Paneth cells and (iii) a decrease in goblet cell number and mucus secretion [15,16]. In addition, HFD induced greater intestinal permeability [15]. A few studies have also investigated the effects of HFD on experimental colitis. Mice receiving HFD were more susceptible to chemically-induced colitis and exhibited more severe colonic inflammation [16,17,18]. Several mechanisms have been suggested: (i) a gut barrier dysfunction, (ii) intestinal hyperpermeability, (iii) pathobiont expansion, and (iv) decreased plasma myokine irisin or adipokine levels [16,17,18]. Nevertheless, effects of HFD are only investigated on acute chemically-induced colitis and their effects on chronic colitis and intestinal fibrosis are still unknown. In fibrosis from extra-intestinal organs, HFD has a deleterious effect. Wnt-β catenin signaling is activated in intestinal fibrosis and can be induced through diet-induced obesity. For example, consumption of HFD induced an upregulation of β catenin and activated epithelial-mesenchymal transition (EMT) in a murine model of colon cancer [19]. This work is in accordance with findings from numerous extra-intestinal models of fibrosis such as hepatic [20] or renal fibrosis [21] where HFD is associated with higher EMT through TGF-β and β catenin signaling.

Environmental factors promoting intestinal fibrosis are currently unknown. High fat diets (HFD) may contribute to promotes fibrosis, as observed in extra-intestinal organs.

HFD have a significant impact on gut barrier function. HFD altered innate immunity response results in: (i) lower production of antimicrobial peptides, (ii) a reduced number of Paneth cells, and (iii) a decrease in goblet cell number and mucus secretion. In addition, HFD induced higher intestinal permeability and HFD-fed mice are more susceptible to colitis. As observed in fibrosis from extra-intestinal organs, HFD may also induce factors that promote intestinal fibrosis such as Wnt-β catenin or epithelial-mesenchymal transition (EMT). A second putative mechanism of HFD on intestinal fibrosis is the involvement of epithelial endoplasmic reticulum stress. Diet is the main modulator of gut microbiota composition. HFD is associated with dysbiosis and may thus impact microbial components. It has been demonstrated that certain bacteria are able to activate ECM production in intestinal fibroblasts. In addition, bacterial ligands are able to promote angiogenesis and it may contribute to fibrosis development. Effects of HFD may be mediated through an increased adiposity. Adiposity may thus play a potential role in initiating and perpetuating intestinal inflammation. Indeed, visceral obesity is associated with higher complications in patients with IBD. Adipocytes are able to secrete adipokines such as leptin or adiponectin. While leptin may exacerbate intestinal fibrosis, adiponectin may exert anti-fibrosis properties through a reduced extracellular matrix (ECM) deposition.

Involvement of epithelial endoplasmic reticulum stress has been recently suggested in CD fibrosis [22] and more precisely HFD is able to exacerbate endoplasmic reticulum stress in a model of pulmonary fibrosis [23].

Adipocytes secrete adipokines such as leptin or adiponectin in IBD mesenteric adipose tissue and serum. While leptin promote a Th1 profile, adiponectin antagonized TNFα and decreased adhesion molecules [24]. Very recently, Xie et al. investigated the effects of intraperitoneal injection of adiponectin in mice with chronic TNBS-induced colitis and they observed that adiponectin treatment reduced inflammatory markers such as colon myeloperoxidase activity and pro-inflammatory cytokines [25]. Adiponectin treatment also reduced extracellular matrix (ECM) deposition. The authors investigated the effect of adiponectin incubation in TGF-β1-treated primary human intestinal fibroblasts and they found that adiponectin reduced collagen level and the phosphorylation of Smad2 [25]. Diet-induced obesity may thus promote intestinal fibrosis via leptin. Other mechanisms may also be involved through microbial components. It has been recently demonstrated that bacterial ligands are able to promote angiogenesis through interaction with CEACAM1 in human intestinal microvascular endothelial cells (HIMEC) [26].

It has been demonstrated that creeping fat are associated to strictures [27]. More recently, Rieder’s team deciphered the mechanisms underlying cross-talks between adipocyte environment from creeping fat and human intestinal muscle cells [1]. Fatty acids derived from creeping fat are able to induce human intestinal muscle cells hyperplasia. In addition, co-culture of HIMEC with whole creeping fat tissue induced their proliferation. They also demonstrated that adipokines from creeping fat of CD patients were able to induce an M2 macrophage subtype and TGF-β, a core cytokine in intestinal fibrosis [28]. Very recently, Devkota’s team also demonstrated that there is specific translocation of a subset of viable bacteria such as *C. innocuum* from the gut microbiota to creeping fat [29]. It limits the systemic dissemination of gut bacteria but also leads to fibrosis development [29]. These mechanisms observed in creeping fat may be also observed from adipocytes from visceral adiposity in obese IBD patients and further studies are required to decipher mechanisms underlying the effects of obesity on complications in IBD patients.

## 3. High Salt

The Western diet is characterized by a high amount of sodium intake >100 mmol/day [30], which is in excess of physiological need (i.e., 10–20 mmol/day). In Western countries processed foods are the main provider of sodium intake (approximately 75% of intake) [30]. A few recent studies have shown the potential of dietary salt to promote intestinal inflammation in colitis models [31,32,33]. It thus raised the potential for dietary salt to induce a more vulnerable environment to inflammatory insults. We have recently demonstrated that a high-salt diet (4%) exacerbates intestinal fibrosis in a rat model of chronic TNBS-induced colitis and fibrosis [34]. We also demonstrated that high salt fed colitic rats had higher undernutrition compared to standard diet fed colitic rats [34]. We investigated the effect of high salt in TGF-β-induced human colon fibroblasts, and reported that NaCl promoted ECM-associated proteins in fibroblasts. Taken together, our study suggested that dietary salt can activate intestinal fibroblasts, thereby contributing to exacerbation of intestinal fibrosis. Further clinical studies are required to investigate whether dietary salt may be considered as a risk factor for intestinal fibrosis.

## 4. High Sugar

Few studies have investigated the effect of sweet diets in colitis models [35,36]. Laffin et al. fed mice with a high sugar diet (50% of sucrose) 2 days before chemically-induced colitis induction. These mice had a higher susceptibility to acute colitis, a higher intestinal permeability, a decreased microbial diversity and a reduced production of short chain fatty acids. In addition, macrophages from high sugar fed mice were more responsive to liposaccharides. Interestingly, the authors were able to reduce high sugar-mediated proinflammatory effects such as histological score or epithelial damage by supplementation with short chain fatty acid acetate in the drinking water [35].

Khan et al. used a different approach by studying the effects of simple sugars in mice. Pre-treatment with simple sugars such as glucose, fructose, or sucrose at 10% in drinking water for 7 days upregulated the histological score and worsened colitis development in chemically-induced colitis development. The authors of the study fed IL-10^−/−^ mice with glucose and this was associated with higher colon inflammatory mediators such as lipocalin-2 or pro-inflammatory cytokines [36]. They also observed a gut dysbiosis in high sugar-diet fed mice, in particular higher abundance of the mucus-degrading bacteria *Akkermansia muciniphila* [36].

The effects of a high sugar diet are not yet documented in preclinical models of intestinal fibrosis. Of note is that glucose is able to induce EMT in many extra-intestinal organs [37,38] and this mechanism may be also relevant in IBD-associated intestinal fibrosis.

## 5. Beneficial Effects of Dietary Components

Contrary to potential deleterious effects mediated by westernized dietary patterns, certain components of diet can prevent intestinal fibrosis development. These nutrients can target several mechanisms involved in intestinal fibrosis. They can act to inhibit or suppress inflammatory processes. They can target specific receptors such as PPARγ or AhR with anti-fibrotic properties. The nutrients can also act at a cellular level by down-regulating EMT processes. Diet is also the main modulator of gut microbiota which may prevent or inhibit fibrogenesis.

### 5.1. Dietary Modulation of Receptors with Anti-Fibrosis Properties

Some nutrients are able to target specific receptors such as PPARγ, AhR, or VDR with anti-fibrotic properties. PPARγ is a nuclear receptor highly expressed in the colon and these anti-fibrotic properties have been investigated by natural and synthetic ligands in IBD models.

AhR belongs to the basic helix–loop–helix superfamily of transcription factors and nutrients such as curcumin or tryptophan metabolites can act as AhR ligands. The AhR is widely expressed in the gut and its activation has been associated with intestinal homeostasis. The role of Vitamin D receptor (VDR) to regulate intestinal inflammation is well documented in preclinical models of IBD. More recently, its involvement in intestinal fibrosis has been reported and invalidation of VDR promotes intestinal fibrosis development in mice in response to DSS-induced colitis.

#### 5.1.1. Peroxisome Proliferator-Activated Receptor γ (PPARγ)

PPARγ is a nuclear receptor highly expressed in the colon and regulates intestinal inflammation [39]. Its anti-fibrotic properties have been investigated in IBD models. Speca et al. have used GED-0507-34, a novel PPARγ agonist, and have shown that preventive PPARγ agonist treatment reduced chronic colitis-induced intestinal fibrosis in mice and ECM-associated factors in TGF-β-induced intestinal fibroblasts and epithelial cells [40].

**Figure 2:**
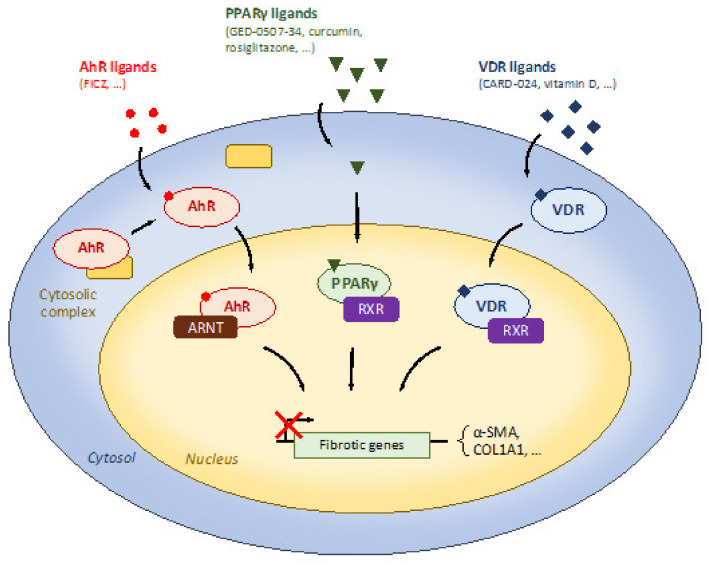
Dietary modulation of receptors involved in the regulation of intestinal fibrosis.

Many nutrients can target PPARγ [39,41] (Figure 2). The natural PPARγ agonist curcumin treatment from 2.5 to 10 μM reduced ECM-associated factors in TGFβ-induced intestinal fibroblasts as can the synthetic PPARγ agonist, rosiglitazone [42]. As myofibroblasts can be derived from various cell types in intestinal fibrosis, the authors validated their findings in epithelial cells and found that curcumin treatment also downregulated TGF-β-associated signaling in intestinal epithelial cells [42]. In addition, this effect was reversed by the use of GW9662, a synthetic PPARγ antagonist, showing the involvement of PPARγ in curcumin-induced anti-fibrotic effects [42]. The authors then confirmed their finding in vivo showing that curcumin at 200 mg/kg reduced chronic colitis-induced intestinal fibrosis and ECM-associated proteins such as fibronectin or CTGF [42]. This is also in accordance with a preclinical study using focal irradiation-induced fibrosis model [43]. In this murine model, 100 mg/kg of curcumin by gavage was able to reduce apoptosis in the injured area and intestinal and plasma IL-6 production [43]. As curcumin use is already validated in UC patients, the authors of this preclinical study hypothesized that curcumin may be relevant as a radioprotector. Novel therapeutic forms of curcumin have been developed, such as polycurcumin [44] or nanoparticle curcumin [45]. Both have been tested in preclinical IBD models and both reduced chemically-induced colitis.

#### 5.1.2. Aryl Hydrocarbon Receptor (AhR)

AhR is a member of the basic helix–loop–helix superfamily of transcription factors, which were first associated with cellular responses to xenobiotics [46,47]. More recently, nutrients such as curcumin or tryptophan metabolites can act as AhR ligands [46,47]. Upon ligand binding, a conformational change leads to AhR translocation into the nucleus and AhR with ARNT heterodimerization to induce target gene expression.

The AhR is widely expressed in the gut and its activation has been associated with intestinal homeostasis regulation [48]. Lamas et al. have shown that treatment with 6-formylindolo(3,2-b) carbazole (FICZ), an AhR agonist, reduced intestinal inflammation in Card9^−/−^ mice [49]. IBD patients exhibited a reduced production of AhR ligands from the gut microbiota [49]. As dietary components can activate AhR to modulate inflammatory responses, Monteleone et al. have investigated whether FICZ exerts anti-fibrotic properties into the gut [50]. From other extra-intestinal fibrotic diseases, dietary ligands of AhR such as 2-(1′H-indole-3′-carbonyl)-thiazole-4-carboxylic acid methyl ester (ITE), L-kynurenin [51] or curcumin [42,52] are able to down-regulate ECM-associated proteins in fibroblasts.

Effects of AhR ligands on IBD-associated intestinal fibrosis are less documented. Treatment with FICZ from 100 to 400 nM decreased ECM-associated gene in stimulated fibroblasts from CD patients [50]. Similarly, 5 ng/mL of TGF-β up-regulated ECM-associated genes such as ACTA2 and COL1A1 in skin fibroblasts while FICZ at 100 nM decreased them [53].

In primary culture of human orbital fibroblasts, 1 ng/mL of TGF-β up-regulated ECM-associated proteins such as fibronectin, collagen I and α-SMA while ITE at 1 μM reduced them [54]. These data are consistent with a study in a liver fibrosis context where ITE treatment at 1 μM for 6 days inhibited ECM-associated proteins such as α-SMA in hepatic stellate cells [55].

#### 5.1.3. Vitamin D Receptor

Epidemiological studies have suggested that low serum vitamin D is associated with an increased IBD risk [56,57]. Similarly, vitamin D and its receptor VDR mediated anti-inflammatory properties in experimental IBD models [58,59]. The role of vitamin D in intestinal fibrosis has been investigated. Johnson et al. have demonstrated that CARD-024, a vitamin D analogue was able to reduce ECM-associated markers in TGF-β-stimulated or stiffness-induced colonic fibroblasts [60]. In addition, down-regulation of colon VDR is observed in chronic CD patients and also in mice with chronic DSS-induced colitis and fibrosis [61]. VDR is also reduced in fibroblasts from CD patients [62]. Mitochondrial dysfunction has been described in patients with IBD and genetic deletion of prohibitin 1, a key protein of the inner mitochondrial membrane decreased in IBD, which can provoke ileitis in mice [63]. VDR is also involved in mitochondrial dysfunction [61] and its specific role in intestinal fibrosis has been recently demonstrated [61]. The authors of this elegant study first demonstrated that VDR expression was lower in intestinal stenotic areas in CD patients [61]. They then induced colitis-induced fibrosis by TNBS or DSS in mice with intestine-specific VDR deletion and they found that VDR deletion exacerbated intestinal fibrosis in both models. To decipher the mechanism underlying these anti-fibrosis effects, they used VDR invalidation in colonic fibroblasts, leading to their activation [61]. VDR invalidation also induced mitochondrial dysfunction mediated epithelial integrity.

### 5.2. Dietary Modulation of Anti-Fibrosis Signaling

Nuclear factor E2-related factor 2 (Nrf2) is a transcription factor involved in anti-oxidant response through the regulation of gene expression. Nrf2 signaling can regulate intestinal inflammation into the gut and has been recently proposed as a putative target in intestinal fibrosis. Nrf2 signaling can be activated by synthetic agonists and nutrients [64]. Sesamin derived from sesame seeds can counterbalance oxidative stress in intestinal epithelial cell line in response to H_2_0_2_ and Nrf2 knockdown abolished the sesamin effect [65]. The authors of this study also evaluated the effects of sesamin at 100 mg/kg in a chemically-induced colitis model and they observed that sesamin was more effective compared to 5-ASA at 50 mg/kg [65]. Other dietary compounds such as numerous polyphenols have been identified to activate NrF2 signaling. Very interestingly, biotransformation of plants by various *lactobacillus* leads to compounds that are dietary ligands of Nrf2 and the Western diet is also characterized by a low consumption of fermented foods compared to our ancient traditional dietary patterns [66].

Some fatty acids derivatives can be partial agonists of cannabinoids receptors (CB1, CB2) agonists and are defined as endocannabinoids. This is the case of anandamide and 2-arachidonylglycerol. By its dual role in intestinal inflammation and metabolic disorders, the endocannabinoid system may be a relevant target in the context of intestinal fibrosis [67]. Indeed, cannabinoid analogues treatment by palmitoylethanolamide (PEA) for 5 weeks was able to counterbalance an ovariectomy-induced mild obesity model with reduced food intake, body weight, and fat mass [68]. Interestingly, PEA treatment also reduced inflammation in colonic biopsies from UC patients and in mice with DSS-induced colitis and these inflammatory effects were mediated through PPARα [69]. This effect has not yet been evaluated in IBD-associated fibrosis but its effect in extra-intestinal fibrosis has been demonstrated [70,71]. Targeting the endocannabinoid system may be particularly useful in the context of obese IBD patients.

### 5.3. Inhibition of Pro-Fibrotic Molecules by Amino Acids

Glutamine is a conditionally essential amino acid [72] and is the preferred fuel used by intestinal cells to promote enterocyte proliferation. Glutamine also regulates tight junctions and reduces proinflammatory signaling [73]. Its effect on preclinical models of intestinal fibrosis has been evaluated. Glutamine enemas at 25 mg/kg reduced colon fibrosis, the number of α-SMA stained cells in the submucosa and ECM-associated proteins in rats with TNBS-induced colitis [74]. These results are in accordance with a study evaluating glutamine treatment in a radiation-induced model where glutamine administration at 1 g/kg/day was able to prevent radiation-induced enteropathy in rats [75]. Similarly, glutamine enemas from 4 to 12 weeks after the surgery reduced colonoscopic and histological scores and reduced the number of collagen fibers in tissue in an experimental model of diversion colitis [76]. Nevertheless, a recent meta-analysis performed on seven published articles about glutamine use in IBD found that glutamine supplementation has no effect on disease course and inflammatory markers in patients with IBD [77] but its effect on fibrosis prevention and/or inhibition has never been evaluated in IBD patients.

Arginine is also a conditionally essential amino acid and we previously demonstrated that arginine treatment was able to down-regulate IL-8 production in cultured intestinal biopsies from CD patients [78]. Nitric oxide is a product of the enzymatic conversion of arginine to citrulline. The role of arginine in intestinal fibrosis is not yet documented but it may be protective through NO pathway. Invalidation of iNOS accelerated high-fat-induced liver fibrosis and inflammation development in mice [79] and this effect was mediated through NO- mediated NF-κB activation. Interestingly, we have shown in an intestinal epithelial cell line that arginine treatment down-regulated cytokines-induced inflammation through the NO pathway [80] and Horowitz et al. have demonstrated that L-arginine treatment up-regulated NO production in HIMEC [81]. Targeting iNOS/NO pathway may be relevant in IBD-associated intestinal fibrosis.

### 5.4. Anti-Fibrosis Properties of n-3 PUFA

We have previously shown anti-inflammatory effects on n-3 PUFA in experimental models of IBD [82] but their effects on intestinal fibrosis are not yet documented. In extra-intestinal organs, n-3 PUFA such as EPA reduced ECM-associated markers and SMAD signaling in TGF-β-induced hepatic stellate cells [83]. These n-3 PUFA effects were reduced by PPARγ knockdown while GW9662, a PPARγ antagonist, did not alter n-3 PUFA effects [83]. In LPS-stimulated dermal fibroblasts, the effects of EPA and DHA were evaluated on fibrosis markers. DHA reduced mRNA levels of α-SMA and collagen III whereas EPA did not. Interestingly, the DHA effect was reinforced when combined with short chain fatty acid butyrate [84]. It is in accordance with a study from Zeng et al., showing that DHA inhibits TGF-β-induced rat renal fibroblast activation at a dose and time-dependent manner [85]. DHA derivative such as resolvin D1 was also evaluated in extra-intestinal fibrosis model [86]. Resolvin D1 treatment was able to reduce mechanical stretch-induced EMT and SMAD signaling in a murine model of lung fibrosis [86].

### 5.5. Dietary Manipulation of the Gut Microbiota

While IBD are strongly associated with shifts in the gut microbiome, the role of microbial factors in intestinal fibrosis is largely unexplored. In vitro, bacterial ligands are able to induce proliferation and migration of intestinal endothelial cells [26]. In chronic DSS, epithelial damage contribute to bacterial translocation and a recent study highlighted the role of flagellin to induce ECM components by intestinal fibroblasts [87]. In vivo, intestinal fibrosis can be abrogated in germ free mice and fibrosis severity is associated with specific microbes in mice overexpressing a member of the TNF superfamilly called TL1a [88]. These specific bacterial strains are able to promote in vitro fibrosis [88]. Imai J et al. have infected mice models with CD-associated pathobiont adherent-invasive Escherichia coli (AIEC). While healthy mice were able to gradually eradicate their infection from the intestine, mice from Salmonella- or DSS-induced colitis models, AIEC infection exploited inflammation to persist leading to intestinal fibrosis through IL-33 receptor signaling [89]. As probiotics strains such as Saccharomyces cerevisiae CNCM I-3856 [90], Lactobacillus, or Bifidobacterium [91] can counterbalance AIEC-promoting inflammation, it may open novel therapeutic avenues in the treatment of intestinal fibrosis.

Diet is a strong modulator of gut microbiota by affecting its composition or as a substrate for microbial production of metabolites. For example, curcumin treatment is also associated with gut microbiota changes. Treatment with nanoparticle curcumin reduced colitis development and increased the abundance of butyrate producing-bacteria and fecal butyrate production [45].

Short chain fatty acids such as butyrate has been evaluated on in vitro angiogenesis in primary cultures of HIMEC [92]. The authors of this study found that butyrate treatment reduced VEGF-induced cellular proliferation, transmigration, and tube formation of HIMEC through down-regulation of COX-2.

### 5.6. Reduction of Myofibroblast Activation

Recently, berberine, an alkaloid extracted from medicinal plants, was able to inhibit EMT [93]. This study used conditioned medium from human intestinal fibroblasts to induce morphological changes and ECM-associated markers in a colonic epithelial cell line and these effects were reversed by berberine treatment at 100 μg/mL for 24 h [93]. The authors demonstrated that berberine reduced EMT by acting on the TGF-β/Smads signaling [93]. Myofibroblasts can be derived from various origins in intestinal fibrosis. For example, endothelial to mesenchymal transition (EndoMT) has been demonstrated in intestinal fibrosis [94]. Nutrients can modify this EndoMT and numerous nutritional approaches have been evaluated. We have previously evaluated DHA, a long chain n-3 PUFA in the primary culture of HIMEC. We have demonstrated that DHA pre-treatment can reduce IL-1β-activated HIMEC pro-inflammatory effects [82] such as decreased adhesion molecule VCAM-1, TLR-4 or cytokine production of IL-6, IL-8. Similarly, curcumin treatment at 10 μM reduced TNF and LPS-induced or irradiation-induced VCAM-1 through NFκB activation in HIMEC [95,96].

### 5.7. Mucosal Healing

The potential effect of probiotics on wound healing have been evaluated. Conditioned medium with the strain *Bacillus polyfermenticus* had pro-angiogenic properties in HIMEC by increasing cell migration, permeability, and tube formation and this effect is mediated though IL-8 production and NF-κB activation [97]. Results were confirmed in vivo in an acute model of DSS colitis [97].

Very few studies have also investigated more complex nutrients compared to unique nutrients in preclinical models of intestinal fibrosis. We have evaluated the effects of a polymeric diet enriched in TGF-β2 in a model of pre-pubertal rats with chronic TNBS-induced colitis and we failed to reverse the inflammation or intestinal fibrosis in our tested conditions [98]. Very recently, a study investigated the effects of fermented rice bran on post-colitis restoration demonstrating a reduction of ECM-associated markers and TGF-β/Smad signaling [99].

## 6. Conclusions

Diet may represent an underestimated risk factor for intestinal fibrosis. A better understanding of the crosstalk between nutrients and factors that promote intestinal fibrosis may enable to provide a better rationale for dietary advice to limit complications in IBD patients. As stipulated by the last ESPEN guidelines for clinical nutrition in IBD, all IBD patients should benefit from dietary counseling by a dietician, which will contribute to limit nutrition-related disorders [100]. In particular, restrictive diets are very popular in IBD patients and are being evaluated in clinical trials and these diets may contribute to a poor psychological well-being [101,102] and lead to undernutrition [100] unless closely supervised. Interestingly, two very recent studies highlighted the potential of Mediterranean diet in IBD patients. While the beneficial effect of this diet has been demonstrated on fibrosis in NAFLD patients with reduced cardiovascular or diabetes risk [103], a 6-month Mediterranean diet was able in IBD patients to reduce malnutrition-associated disorders, improved disease activity and inflammatory markers with a concomitant increased of a quality of life score [104]. The need for further nutritional intervention trials with diets that target factors-promoting intestinal fibrosis and/or address the Westernization of food in IBD-associated intestinal fibrosis are urgently required.

## Data Availability

Not applicable.

## Abbreviations

CD: Crohn’s disease; ECM, extracellular matrix, EMT, epithelial-mesenchymal transition; IBD, inflammatory bowel disease; Nrf2, Nuclear factor E2-related factor 2, PPARγ, Peroxisome Proliferator-activated Receptor γ, UC, ulcerative colitis.
